# Effects of Slightly Acidic Electrolyzed Water on the Quality of Fresh-Cut Apple

**DOI:** 10.3390/foods12010039

**Published:** 2022-12-22

**Authors:** Qing Gao, Ziyi Yang, Baoliang Bi, Jinsong He

**Affiliations:** 1 College of Food Science and Technology, Yunnan Agricultural University, Kunming 650201, China; 2 International College, Yunnan Agricultural University, Kunming 650201, China

**Keywords:** slightly acidic electrolyzed water, fresh-cut fruit, storage quality

## Abstract

To investigate the effects of a slightly acidic electrolyzed water (SAEW) treatment on the quality of fresh-cut apples during storage, this research used a Box–Behnken design to determine the optimal SAEW treatment conditions. Then, the fresh-cut apple was treated under the optimal condition and subjected to a 13-d storage experiment at 4 °C. For fresh-cut apple treated under the optimal SAEW treatment conditions, the total number of surface colonies was reduced by 2.82 logarithms compared to the control group and the sensory score was 8.73. For the treated fresh-cut apple during storage, the quality of the treated group was significantly greater than the non-treatment group. Thus, the SAEW treatment not only effectively controlled the number of microbes on fresh-cut apple, but also slowed quality deterioration during storage.

## 1. Introduction

As a specialty fruit, apple has the characteristics of high yield, bright color, crispy pulp, balanced sweetness and sourness, and rich flavor. It is suitable for direct consumption. With the accelerated pace of living, fresh-cut fruits are gaining popularity with more consumers and are gradually becoming a main source of fruit consumption. Fresh-cut fruit refers to processed fruit products, which are consumed instantly by consumers or the catering industry, after being sorted, rinsed, peeled, trimmed, cut, and packaged [1]. Because of their characteristic freshness, health benefits, cleanness, and convenience, they have become one of the fastest growing categories of retail foods [2]. Therefore, the development of fresh-cut apple products needs to meet the demands of modern populations, but they also need variety, added value, and to extend the industrial chain.

Microbes are a main factor affecting the storage quality of fresh-cut fruit during transportation and retail. Mechanical damage to the cells and tissues during peeling and cutting processes can result in microbial infection, physiological aging, nutrient loss, tissue discoloration, texture softening or lignification, and flavor deterioration, which affect storage quality and shorten shelf life [3]. Currently, peroxyacetic acid, ozone, sodium hypochlorite, acidified sodium chlorite, chitosan, antibiotics, and spice extracts have been applied during fresh-cut fruit and vegetable processing, but they all impact the safety, quality, and flavor of the fresh-cut products [4,5].

To control the number of microbes on fresh-cut fruit, scholars within China and worldwide have discussed the application of non-thermal processing technology, because the traditional thermal sterilization technology is not suitable. For instance, non-thermal processing by ultrasound treatment of fresh-cut quince slices inhibited the growth of bacteria, mold, and yeast, compared with hot water treatments, and also improved their physical properties when stored at 4 °C [6].

The application of slightly acidic electrolyzed water (SAEW) is a non-thermal sterilization technique. Based on different pH values, available chlorine concentration (ACC), and oxidation-reduction potential (ORP), acidic electrolyzed water can be categorized into strongly acidic electrolyzed water (pH < 2.7; ACC, 20–200 mg/L; ORP, 900–1200 mV), weakly acidic electrolyzed water (pH 2.7–5.0; ACC, 20–60 mg/L), and SAEW (pH 5.0–6.5; ACC, 10–90 mg/L; ORP 700–900 mV). In the SAEW pH range, more than 90% of the available chlorine is in the form of hypochlorous acid, which has a bactericidal activity 80–150 times that of hypochlorite. Therefore, SAEW has relatively high bactericidal activity at low ACCs. To date, it has been applied in environmental, medical [7], and agricultural fields [8,9,10,11].

SAEW is characterized by the absence of a residue, its easy preparation, and innocuousness to the human body [12,13,14]. It was approved as a permitted food additive by the Ministry of Health, Labor, and Welfare of Japan, and is currently being used in the food industry [15,16,17]. The effectiveness of SAEW as a bactericide and the optimal conditions for pathogens have been studied in several reports. Using a mixed-species biofilm of Lis-teria monocytogenes Scott A and Staphylococcus aureus, the researchers examined SAEW’s disinfectant properties after 12 months. The result shows bacillus cereus, a spore-forming pathogen, was completely killed by SAEW at 30 and 50 ppm within 30 s [18]. Moreover, an analysis of pH, available chlorine concentration (ACC), and oxidation-reduction potential (ORP) of SAEW samples collected after sterilizing fresh cabbage with SAEW solution was carried out in another study to determine the antiviral effectiveness of SAEW. After evaluating the effect of SAEW on the inhibition of bacterial growth of fresh-cut cabbage and confirming that SAEW solutions decay after disinfection, a kinetic model was developed [19]. To date, research on the application of SAEW in the field of fresh-cut food has mainly focused on the bactericidal effects on surface microbes, but there are relatively few reports on the optimization of treatment conditions and effects on storage quality.

In summary, this study investigated the optimal SAEW treatment conditions and the effects on the storage quality of fresh-cut apples, to explore the prospects of applying SAEW to control microbial contamination and mitigate the deterioration of the storage quality of fresh-cut fruit.

## 2. Materials and Methods

### 2.1. Materials

Apples were purchased from local orchards of Zhaotong, China, and were of the same maturity level and without pests, disease, or mechanical damage. Sulfuric acid (81646-13-1), oxalic acid (144-62-7), phenol (591-27-5), sodium chloride (7647-14-5), vitamin C (50-81-7), glucose (58367-01-4), and soluble starch (9005-84-9) were purchased from Sichuan Xilong Chemical Co., Ltd., Chengdu, Sichuan. Plate count agar (9002-18-0) medium was purchased for the mass concentration iodometric method from Guangdong Huankai Microbiology Technology Co., Ltd., Guangdong, China. All the reagents used for buffer solutions were of analytical grade.

### 2.2. SAEW Preparation and Characterization of Physical and Chemical Properties

SAEW was generated by the “Water God” SAEW generator (HD-240L, Shanghai Want Want Group) to electrolyze a dilute hydrochloric acid solution (mass concentration of 9%). The equipment was running for 30 min. After the available chlorine had stabilized, SAEW was collected for testing and used within 1 h after preparation [14].

The pH value and redox potential of the electrolyzed water samples were determined using a SevenMulti pH/conductivity/ion modular meter system (Shanghai Mettler Toledo Instrument Co., Ltd., Shanghai, China), and the mass concentration of available chlorine was determined using the iodometric method [20].

### 2.3. Processing of Raw Material

The apple samples were rinsed with tap water for one minute, and then they were placed on a bench that had been sterilized with ultraviolet light. Preparing the apple samples for testing involved peeling them with a fruit knife that has been sterilized with 75% ethanol, removing the seeds from each apple sample, cutting each sample up into uniform squares measuring 2 cm by 2cm by 1 cm, and weighing each sample at 5 g.

### 2.4. Optimization of SAEW Treatment Conditions

The fresh-cut apples were treated with SAEW under different conditions. Using microbial lethality as the benchmark, single factor experiments were performed with the variables ACC, treatment time, and liquid-to-material ratio. Each experiment was repeated three times, with 50-g samples. When the treatment time was 3 min and the liquid-to-material ratio was 8:1 mL/g, the ACCs were10, 20, 30, and 40 mg/L. When the ACC was 20 mg/L and the liquid-to-material ratio was 8:1 mL/g, the treatment times were 1, 3, 5, and 7 min. When the ACC was 20 mg/L and the treatment time was 3 min, the liquid-to-material ratios were 4:1, 8:1, 12:1, and 16:1 mL/g.

Based on the single factor experiments, a model was established by a Box–Behnken design. The optimization of conditions was performed using the ACC, treatment time, and liquid-to-material ratio of the SAEW as the main independent variables (A, B, and C, respectively), and microbial lethality rate and sensory score as the dependent values (*Y*_1_ and *Y*_2_). The high, medium, and low levels of the independent variables were represented by +1, 0, and −1, respectively. The codes for variables and their levels are shown in Table 1.

For the microbiological examination, the procedures of “Food Microbiological Examination: Aerobic Plate Count” were used. The total number of surface colonies on the sample was determined, and the bactericidal effect was [21] represented by the microbial lethality rate *Y*_1_. The calculation method was as follows:

(1) $$Y_{1}=\mathrm{lg}\left(\frac{N_{0}}{N}\right)$$

where *N*_0_ represents the total number of surface colonies on the untreated sample (CFU/g); and *N* represents the total number of surface colonies on the SAEW-treated sample (CFU/g).

Specifically, the taste evaluation (total 5.5 points) includes crisp flesh, perfect sweetness and sourness, rich taste, and no smell. When fresh-cut apple examples failed to meet expectations for each criterion, half or one point was docked. Regarding the color determination part (total 3 points), any fresh-cut apple examples were discounted because of the browning ratio. The browning ratio will lead to a deduction of up to one, two, or three points if it is below 30%, between 30% and 80%, and above 80%, respectively. As a hardness evaluation part (total 1.5 points), the firmness and freshness of the flesh in the fresh-cut apple example had to match. The firmness score was deducted by one point or one and a half points if more than half of the flesh was soft and almost inedible [21].

### 2.5. Analysis of Sample Quality during Storage

Using the optimal treatment conditions obtained from optimization, the fresh-cut apples were treated with SAEW, drained for 10 min, packaged with 0.04-mm thick polyethylene plastic film, and stored in a 4 °C cold room for 13 d. The total number of colonies, vitamin C content, color, total sugar content, weight loss rate, and pH were measured periodically. Samples treated with sterile water were used as the control group.

As part of the homogenizing process, a 100 g apple sample was homogenized in 300 mL of pure water at 19,000 rpm for 2 min using the Polytron ST20 apparatus (Kinematika, Lucerne, Switzerland) equipped with OD-S shafts (20 mm in diameter), which was then centrifuged for a period of 15 min at 10,000 rpm, and the supernate was collected. To determine the vitamin C content, the iodometric method was used [22]. Twenty mL of supernate was placed in an Erlenmeyer flask, and 1 mL of 2% starch solution was added. The solution was titrated with an I_2_ standard solution until a light blue color appeared and persisted for 30 s. The titration values were recorded.

Determination of total sugar content: phenol–sulfuric acid colorimetric method [23] was used. One mL of supernate was placed in a test tube, to which 1 mL of pure water, 1 mL of phenol, and 5 mL of concentrated sulfuric acid were added. The mixture was shaken and allowed to stand for 30 min. The absorbance at 620 nm was measured and recorded, and the total sugar content was calculated.

To determine the pH value, 20 mL of supernate was placed in a small beaker, and the pH value was measured using a pH meter and recorded.

The weight loss rate was determined by the weighing method [24]. Briefly, 100 g of apple sample was weighed by an electronic balance, bagged, and stored. The weight was recorded at each examination, and the results were expressed as the difference between the initial fruit mass and the mass at each measurement as a percentage of the initial fruit mass.

A colorimeter was periodically used to measure the color of fresh-cut fruit surfaces. Apple samples were collected from both the SAEW group and the control group in order to make comparisons between them. The colorimeter records each value of *L*, *a*, and *b* for each color. This formula can be used to calculate the total brightness change ∆*E* with the following formula [23]:

(2) $$\Delta E=\sqrt{(L_{0}-L)+{\left(a_{0}-a\right)}^{2}+{\left(b_{0}-b\right)}^{2}}$$

where *L*_0_, *a*_0_, *b*_0_ represents standard color and *L*, *a*, *b* represents measured color.

### 2.6. Statistical Analyses

Design-Expert (Ver. 7.0.0, Stat-Ease) was used for the optimization process, Model Graph software was used for plotting response surface graphs, a numerical analysis of the optimization program was used to predict the optimal values. Excel (ver. 2003, Microsoft) was used for data analysis. Origin (Ver. 8.0, Origin Lab) was used for chart processing. SPSS (ver. 18.0, SPSS) was used for the significance analysis, with the significance level set at *p* = 0.05.

## 3. Results and Discussion

### 3.1. Optimization of SAEW Treatment Conditions

#### 3.1.1. Effects of Different Treatment Variables

**Figure 1:**
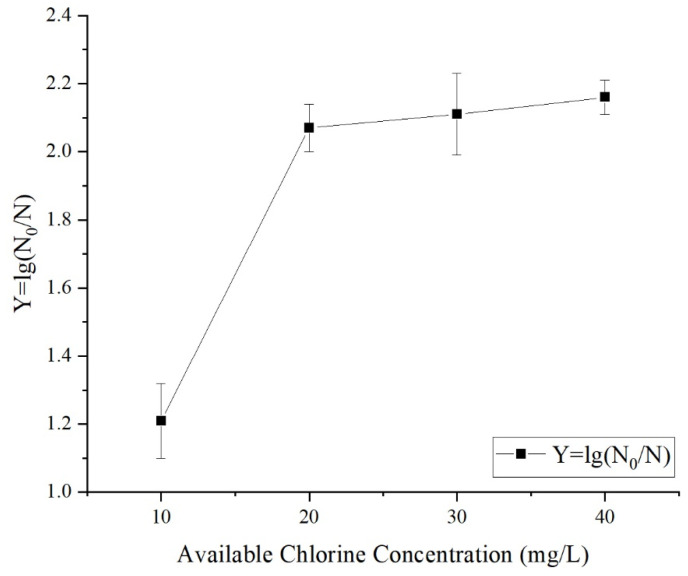
Effect of different concentrations of acc on sanitizing effect in fresh-cut apple.

To investigate the effects of the SAEW ACC’s bactericidal capability on fresh-cut apple, fresh-cut samples were mixed with SAEW with different ACCs (10, 20, 30, and 40 mg/L) with a liquid-to-material ratio of 8:1 mL/g. After soaking for 3 min, the total number of surface colonies was determined, and the microbial lethality rate was calculated. As shown in Figure 1, the SAEW had a significant bactericidal effect on the surface microbes present on fresh-cut apple. As the ACC, the bactericidal effect increased, and the lethality rate increased significantly (*p* < 0.05) from 1.21 logarithms at 10 mg/L to 2.07 logarithms at 20 mg/L. When the liquid-to-material ratio was 20–40 mg/L, the microbial lethality rate tended to plateau, and the bactericidal effect did not display a noticeable change (*p* > 0.05).

**Figure 2:**
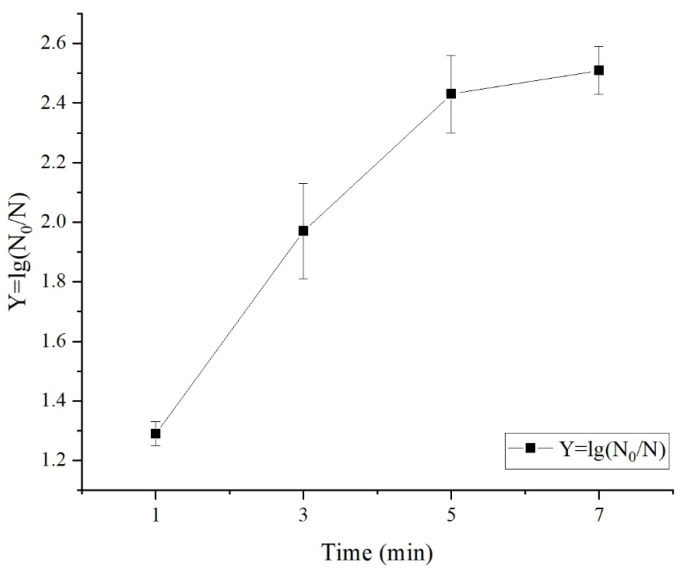
Effect of Different Processing Time on Sanitizing Effect in Fresh-cut Apple.

To evaluate the effect of treatment time on the bactericidal capability, fresh-cut apple was soaked for different durations (1, 3, 5, and 7 min) in an SAEW with an ACC of 20 mg/L and a liquid-to-material ratio of 8:1 mL/g. As shown in Figure 2, the lethality rate of microbes on the surface of fresh-cut apple increased significantly (*p* < 0.05) as the treatment time was prolonged, from 1.29 logarithms at 1 min to 2.43 logarithms at 5 min. After the treatment time reached 5 min, no significant changes in the total number of colonies or the lethality rate were observed (*p* > 0.05).

**Figure 3:**
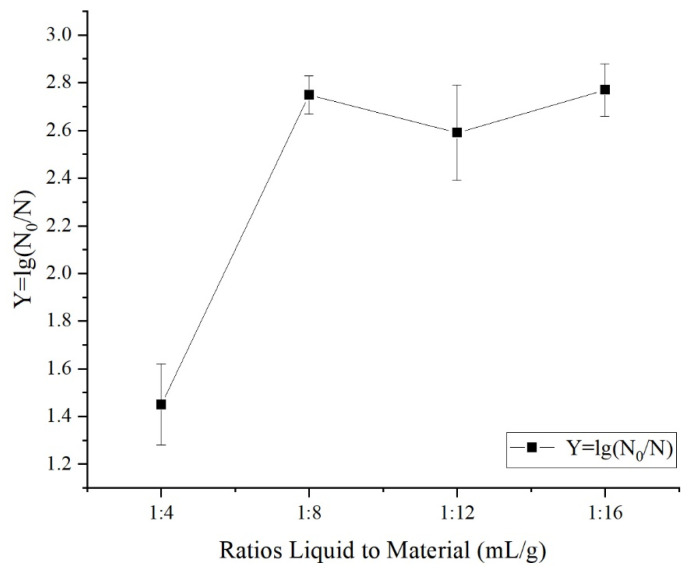
Effect of different ratios of liquid to material on sanitizing effect in fresh-cut apple.

To investigate the effect of the liquid-to-material ratio on the bactericidal capability, the samples were treated with an ACC of 20 mg/L and liquid-to-material ratios of 4:1, 8:1, 12:1, and 16:1 mL/g for 5 min of treatment. As shown in Figure 3, as the liquid-to-material ratio increased, the lethality rate of surface microbes on fresh-cut apples first increased and then plateaued. The mortality rate increased from 1.45 logarithms at 4:1 mL/g to 2.75 logarithms at 8:1 mL/g (*p* < 0.05). Then, the surface microbial mortality rate did not change appreciably as the liquid-to-material ratio increased (*p* > 0.05).

#### 3.1.2. Establishment of the Regression Equation

To ensure that the SAEW treatment effectively controlled microbial growth on fresh-cut fruit and did not compromise the sensory qualities of the fruit, the Design-Expert 7.0 software was used for optimization experiments based on the results of the single-factor experiments. The design and results are shown in Table 2.

Multivariate regression fitting was performed with the data in Table 3, and the quadratic multiple regression equations of dependent values, *Y*_1_ (microbial lethality rate) and *Y*_2_ (sensory score), and the independent variables (*A*, *B*, and *C*) were obtained (variables were the coded values) as follows:

(3) $$Y_{1}=3.20+1.05A+0.35B+0.11C+0.04AB+0.09BC-0.08AC\;-0.49A^{2}-0.34B^{2}-0.25CA^{2}$$

(4) $$Y_{2}=8.98-0.38A-0.11B+0.08C+0.04AB-0.01BC-0.04AC\;-0.79A^{2}-0.29B^{2}-0.19C^{2}$$

To verify the accuracy levels of the regression equations, the model was subjected to a variance analysis (Table 4). As shown in Table 4, the *p* values of regression Equation (2) and the model were <0.0001, indicating that the model is highly significant. The lack of fit (*F* = 0.10, *p* = 0.955 > 0.05) and the difference were not significant, indicating that the residual was the result of random error. The coefficient of determination R^2^ = 0.957 and the model-adjusted coefficient of determination R_adj_^2^ = 0.902, indicating that the fit is good, the model can account for changes in the dependent values, the experimental error is small, and the model can be used to analyze and predict the microbial lethality rate.

The *p* values of regression Equation (3) and the model were <0.0001, indicating that the model is highly significant. The lack of fit (*F* = 2.03, *p* = 0.253 > 0.05) and the difference were not significant, indicating that the residual was the result of random error. The coefficient of determination R^2^ = 0.999 and the model-adjusted coefficient of determination R_adj_^2^ = 0.999, indicating that the fit is very good, the model can account for the changes in the dependent values, the experimental error is small, and the model can be used to analyze and predict the sensory scores of the samples.

Based on the above results, the relationships between the three independent variables (A, B, and C) and the two dependent values (*Y*_1_ and *Y*_2_) were validated. Thus, the regression model could be used instead of actual experimental data points to analyze the experimental results.

To determine the main effect order of different variables on the treatment, the regression coefficients in the model were subjected to a significance analysis. As shown in Table 5, for the dependent value *Y*_1_, the primary terms A (ACC, *p* < 0.0001), B (treatment time, *p*< 0.0001), and C (liquid-to-material ratio, *p* < 0.05) had significant effects; the quadratic term C^2^ (*p* < 0.05) had significant effects; the interaction terms AB, AC, and BC (*p* < 0.01) had highly significant effects. For the dependent value *Y*_2_, the effects of the primary terms A, B, and C (*p* < 0.0001) were highly significant; the effects of the quadratic terms A^2^, B^2^, and C^2^ (*p*< 0.0001) were highly significant; the effects of the interaction terms AB and AC (*p* < 0.01) were highly significant. Thus, the effects of the variables on the dependent values *Y*_1_ and *Y*_2_ were quadratic. The absolute value of each coefficient in the equations directly reflects the extent of the influence of each variable on the response value, and the positive or negative signs of the coefficients reflect the direction of influence. Based on estimated values of the primary term coefficients, the main effect order of the independent variables was: A > B > C.

#### 3.1.3. Response Surface Interaction Analysis

Because the single-factor experiments only reflect the impact of a single variable on the results, the optimal combination of variables and the optimal dependent values in the whole region could not be determined. Therefore, in order to explore the SAEW treatment’s effects on the microbial population and sensory qualities of the samples more accurately, a response surface analysis of the interactions between different variables was required.

**Figure 4:**
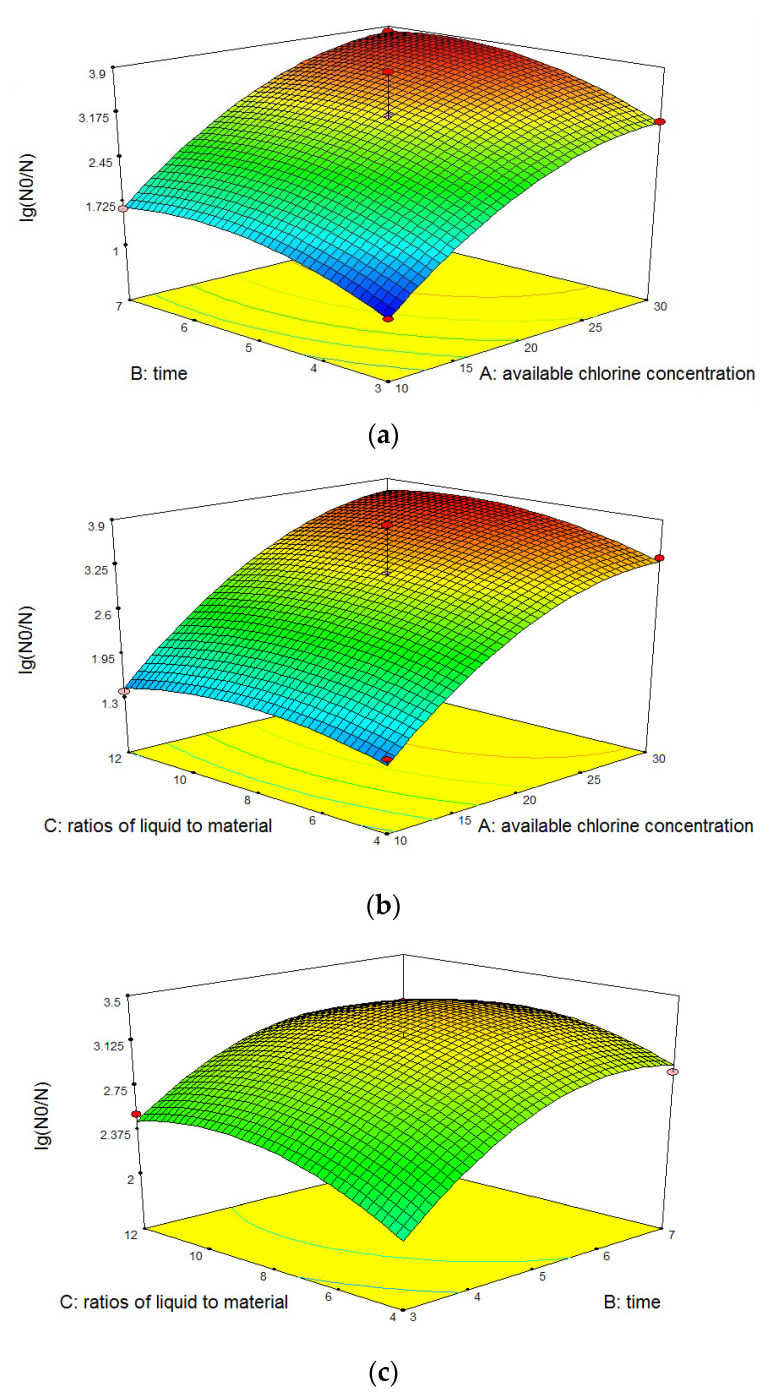
(**a**). Response surface of Y = f_2_(A,B). (**b**). Response surface of Y = f_2_(A,C). (**c**). Response surface of Y = f_2_(B,C). Response surface of sensory evaluation treated with SAEW on fresh-cut apple.

The slope of the response surface reflects the sensitivities of the dependent values to changes in the independent variables. As shown in Figure 4a, within the range of designed experimental conditions, as the ACC or treatment time increases, the dependent value increases gradually. The slope of the response surface was steep, indicating that the microbial lethality rate was susceptible to influences of ACCs and treatment times. As shown in Figure 4b, the microbial lethality rate was relatively sensitive to the ACC. As the ACC increases, the dependent value increased rapidly and then plateaued. The microbial lethality rate was less sensitive to the liquid-to-material ratio, and the dependent value increased slowly as the liquid-to-material ratio increases. As shown in Figure 4c, as the treatment time and liquid-to-material ratio increased, the dependent value increased slowly, and the slopes were more gradual, indicating that the microbial lethality rate was less sensitive to the treatment times and liquid-to-material ratios. The trends of response surfaces were, in general, consistent with the results of the single-factor experiments. These results, combined with the significance analysis results of the regression coefficients’ interaction terms (Table 5), in which the *p* values of the AB, AC, and BC were all greater than 0.05, indicated that the interactions among these three variables were not significant.

**Figure 5:**
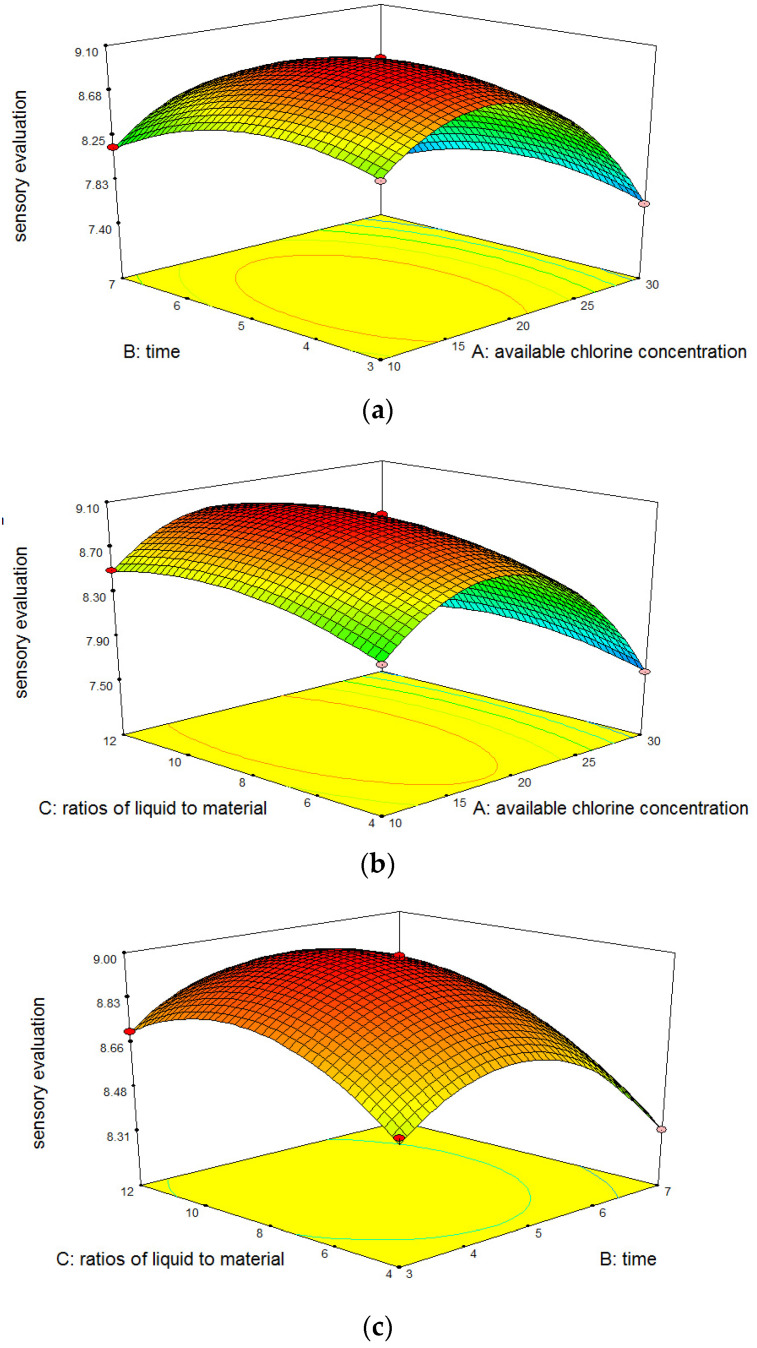
(**a**). Response surface of Y = f_2_(A,B). (**b**). Response surface of Y = f_2_(A,C). (**c**). Response surface of Y = f_2_(B,C). Response surface of sensory evaluation treated with SAEW on fresh-cut apple.

As shown in Figure 5a, as the ACC and treatment time increased, the sensory scores of the apple samples first increased and then decreased. When the ACC was 20 mg/L and the treatment time was ~5 min, the sensory score reached its maximum value. As shown in Figure 5b, as the ACC and liquid-to-material ratio increased, the sensory scores first increased and then decreased, and the ACC exerted a significantly stronger influence than the liquid-to-material ratio. As shown in Figure 5c, as the treatment times and liquid-to-material ratios increased, the sensory scores gradually increased. However, after the treatment time exceeded 5 min and the liquid-to-material ratio was greater than 8:1 mL/g, the sensory scores declined rapidly. As shown in Figure 5, certain interactions exist among ACC, treatment time, and liquid-to-material ratio, and the sensory quality of fresh-cut apple was adversely affected if any of the three variables was too high or too low. Combined with the coefficients of the interaction terms in Table 5, AB, AC (*p* < 0.01), and BC (*p* > 0.05), it was concluded that significant interactions existed between the ACC and both the treatment time and the liquid-to-material ratio.

#### 3.1.4. Determination and Verification of the Optimal Treatment Conditions

To maintain the bactericidal effects without greatly affecting the quality of fresh-cut apple, the optimal SAEW treatment conditions were determined. The two dependent values, microbial lethality rate and overall sensory score, were used. The goal criteria were set at the maximum values, and a numerical analysis was performed. Based on the relevant experimental data (Table 3), the overall optimal ACC was 21.27 mg/L, the optimal treatment time was 5.20 min, and the optimal liquid-to-material ratio was 8.85:1 mL/g. For ease of operation, the conditions were adjusted as follows: the ACC was set to 21 mg/L, the treatment time to 5 min, and the liquid-to-material ratio to 9:1 mL/g.

To verify the accuracy of the optimized conditions, fresh-cut apple was treated under these conditions, and three sets of validation experiments were performed, with microbial lethality rates and sensory scores as the verification indicators.

As shown in Table 5, the actual measured microbial mortality rate was 3.25 ± 0.32 lg (CFU/g), and the actual measured sensory score was 8.73 ± 0.08, which were largely consistent with the predicted values, indicating that the optimized conditions were reasonably reliable.

### 3.2. Analysis of Quality Changes during Storage

To assess the effects of the SAEW treatment on changes in the quality of fresh-cut apple samples during storage under the above optimal conditions, the apple samples were soaked in the SAEW (ACC = 21 mg/L, pH = 6.06) for 5 min at a 9:1 mL/g liquid-to-material ratio, and the storage test was carried out at 4 °C.

#### 3.2.1. Effects of the SAEW Treatment on Total Number of Colonies

Damage to fresh-cut apple surfaces facilitates microbial growth, and microbial contamination and proliferation cause a deterioration in the quality of fresh-cut fruit. In the storage experiments, the effects of the SAEW treatment on microbes were studied.

**Figure 6:**
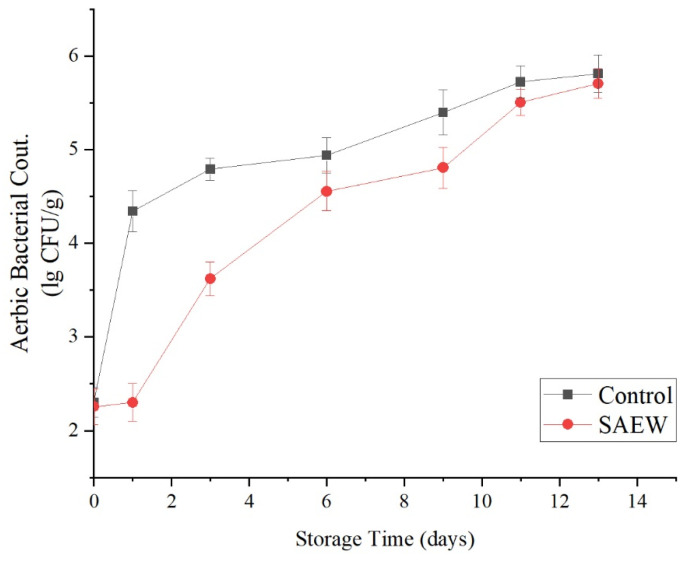
Effect of changes of colonies number on sample of apples treated with SAEW (4 °C).

As shown in Figure 6, in both the SAEW-treated group and the sterile water-treated control group, the total numbers of colonies on the surfaces of fresh-cut products increased with storage time. However, the rate of microbial growth on the SAEW-treated group was markedly slower than that on the control group, and the total amount of microbes in the SAEW-treated group was significantly less than that of the control group after the same number of days in storage. From d 0 to d 9, the total numbers of surface colonies on the SAEW-treated group and the sterile water-treated group increased from 0.62 lg (CFU/g) and 2.30 lg (CFU/g), respectively, to 4.81 lg (CFU/g) and 5.40 lg (CFU/g), respectively. The two groups exhibited significant differences (*p* < 0.01). However, the difference between the two groups became insignificant after 11 d of storage (*p* > 0.05). At the end of storage, the total numbers of colonies had reached 5.71 lg (CFU/g) and 5.81 lg (CFU/g), respectively.

Thus, the SAEW treatment effectively inhibited microbial growth on fresh-cut apple during storage, and maintained a good inhibitory effect for one week. Under slightly acidic conditions, the available chlorine is mainly present in the form of hypochlorous acid, which increases the permeability of microbial cell membranes, resulting in microbial growth inhibition and death within a certain time range [25].

#### 3.2.2. Effects of SAEW Treatments on the Vitamin C Content of Fresh-Cut Apple Samples

Vitamin C is easily lost during owing to storage temperature, time, and mechanical damage. The loss of Vitamin C is a main factor affecting the nutritional value of fresh-cut fruit.

**Figure 7:**
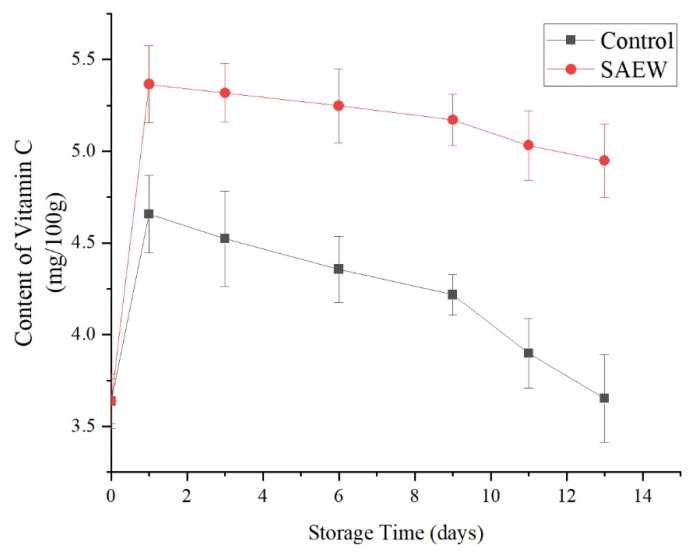
Effect of changes of content of VC on sample of apples treated with SAEW (4 °C).

As shown in Figure 7, in both the SAEW- and the sterile water-treated groups, the vitamin C content of the samples initially increased and then decreased with storage time, reaching maximum values on the first day and then gradually declining. However, over the same storage time, the vitamin C content of the SAEW-treated group was greater than that of the sterile-water treated group (*p* < 0.05). On the 13th d of storage, the vitamin C content of the SAEW-treated group decreased from 5.37 mg/100 g on the first day to 4.95 mg/100 g, a decline of 7.82% from the maximum value, while the vitamin C content of the control group decreased from 4.66 mg/100 g on the first day to 3.65 mg/100 g, a decline of 21.67% from the maximum value.

These results indicated that the SAEW treatment suppressed the decrease in the vitamin C content of fresh-cut apple. This may be attributed to the SAEW treatments’ inhibition of microbial growth (Figure 6) and some enzyme activities, which slow the loss of nutrients in fresh-cut fruit [26], including vitamin C. The vitamin C content of some fruits, such as cantaloupe, is less affected by storage time. There was no significant change in vitamin C content in fresh-cut cantaloupes until day nine [27]. At the 10th day, the vitamin C content of fresh-cut apple treated with weakly acidic electrolyzed water was significantly greater than strongly acidic electrolyzed water, or sterile water. These findings are consistent with the results of the present study.

In addition, the finding that the vitamin C contents of the samples reached peak values on the first day may be the result of mechanical damage caused by the fresh-cut treatment, which promotes the synthesis of ethylene in the fruit and activates phenylalanine ammonia-lyase, which causes a rapid increase in the vitamin C content [28].

#### 3.2.3. Effect of an SAEW Treatment on the Color of Fresh-Cut Samples

Color is an important indicator of fresh-cut fruit. The ∆*E* value reflects the brightness of food, and the color change process of fruit is described using ∆*E* values.

**Figure 8:**
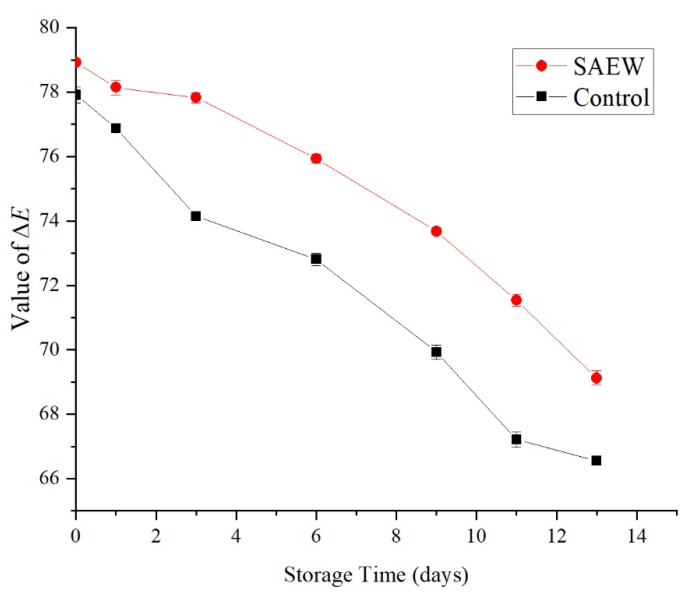
Effect of color on sample of apples treated with SAEW (4 °C).

As shown in Figure 8, the brightness ∆*E* values of both the SAEW-treated and the sterile water-treated control groups decreased with the extension of storage time, indicating that the browning of fresh-cut apple increased with time. Although no significant difference in ∆*E* values was observed between the SAEW-treated group and the control group at 1 d after treatment (*p* > 0.05), after 1 d of storage, the decrease in the ∆*E* value of the SAEW-treated group was markedly slower than that of the control group, and the ∆*E* value of the SAEW-treated group was significantly greater than that of the control group (*p* < 0.05) for the same storage time. After 13 d of storage, the ∆*E* value of the SAEW-treated group had decreased from 78.92 (at the beginning of storage) to 69.12, while the ∆*E* value of the control group decreased from 77.91 (at the beginning of storage) to 66.56.

The above results demonstrated that the SAEW treatment inhibited the color change of fresh-cut apple, thus slowing the browning process and exerting a certain protective effect on the color. Polyphenol oxidase is widely regarded as the key enzyme responsible for the color change of fruit and vegetables. Ling et al. reported that weakly acidic electrolyzed water reduces the activity of polyphenol oxidase, thus inhibiting the browning of *Zizania latifolia* [29]. In addition, the antioxidant activity of vitamin C may be responsible for the significant less-pronounced color change in fruit treated with SAEW compared to fruit treated with sterile water (Figure 7).

#### 3.2.4. Effects of the SAEW Treatment on the Total Sugar Content of Fresh-Cut Apple

The total sugar content influences the color, smell, taste, tissue morphology, and nutritional value of fresh-cut fruit products. Meanwhile, it is an important indicator of the quality change in fresh-cut apple during storage.

**Figure 9:**
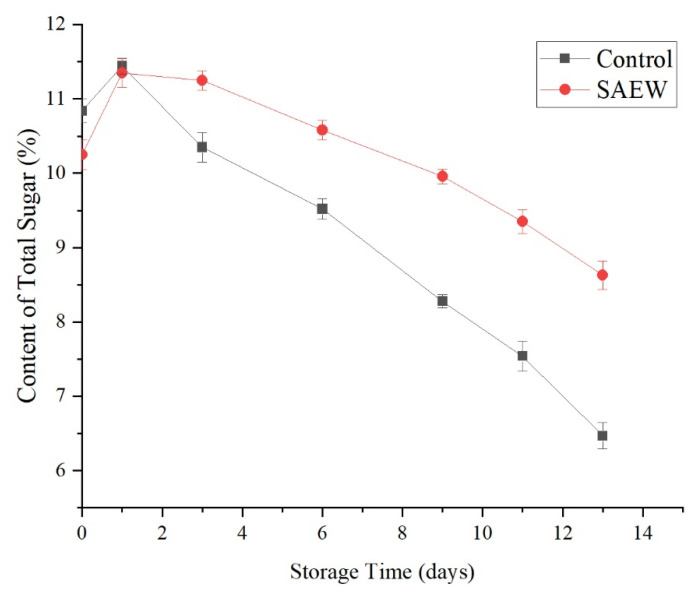
Effect of changes of total sugar on sample of apples treated with SAEW (4 °C).

As illustrated in Figure 9, the total sugar content in the SAEW-treated and the sterile water-treated control groups continuously decreased as the storage time increased. However, except for the first day, the total sugar content of the SAEW-treated group was significantly greater than that of the control group (*p* < 0.01). On d 13 of storage, the total sugar content of the SAEW-treated group was 8.63%, whereas the total sugar content of the control group was only 6.47%.

Thus, the SAEW treatment appeared to inhibit the decrease in the total sugar content of fresh-cut apple during storage. The SAEW treatment slowed the decrease in the total sugar content of fresh-cut apple, likely because it not only inhibited microbial growth, but also because it maintained the respiratory activities of the samples at relatively low levels, thus mitigating the decline in the total sugar content.

In addition, because the mechanical damage of the fruit generates ethylene, which stimulates fruit respiration and accelerates the fruit ripening process [30], the total sugar content increased on the first day.

#### 3.2.5. Effects of the SAEW Treatment on the Weight Loss Rate of Fresh-Cut Apple

Owing to respiration and transpiration, fresh-cut apple is highly prone to water loss, which affects its freshness. Therefore, the weight loss rate is an important indicator of fresh-cut fruit quality.

**Figure 10:**
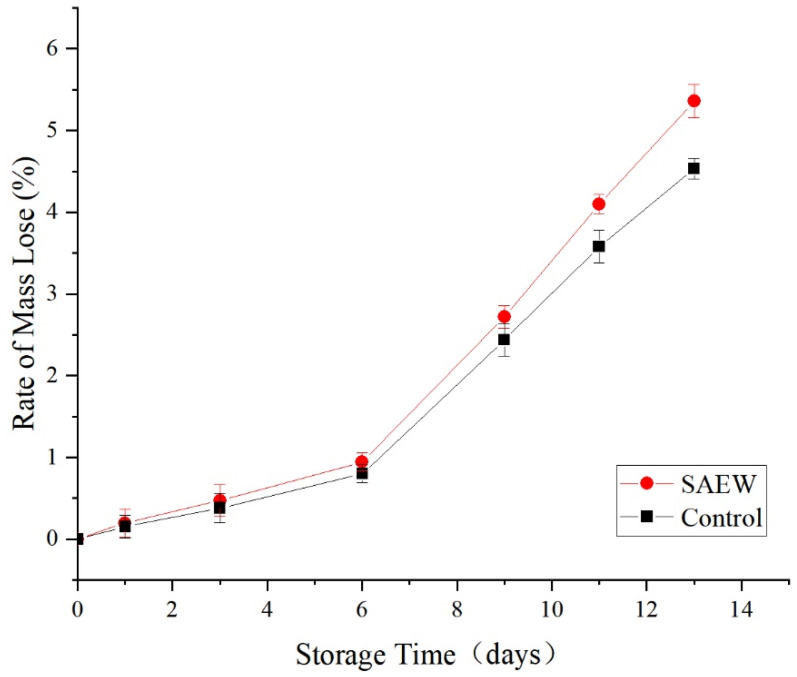
Effect of changes of mass loss rate on sample of apples treated with SAEW (4 °C).

As shown in Figure 10, the weight loss rate of both the SAEW-treated and the sterile water-treated groups increased with prolonged storage times. The weight loss rates of the SAEW-treated and control groups were relatively slow within the first 6 d of storage but began to increase rapidly on the d 6. Based on comparisons, no significant differences in weight loss rates between the SAEW-treated and the sterile water-treated samples were observed during the first 9th d of storage (*p* > 0.05). Afterwards, the weight loss rate of the SAEW-treated group was significantly lower than that of the control group (*p* < 0.05). On d 13 of storage, the weight loss rates of the SAEW-treated and the control groups were 4.53% and 5.36%, respectively.

Thus, the SAEW treatment alleviated the weight loss of fresh-cut apple. The weight loss rate of the control group was significantly greater than that of the weakly acidic electrolyzed water-treated, the strongly acidic electrolyzed water-treated, and the sodium hypochlorite-treated groups. The weight loss rate of fresh-cut lotus root was lowest in the weakly acidic electrolyzed water-treated group [21]. The loss of water and consumption of nutrients are the main paths through which fruit quality is compromised. The SAEW treatment reduces the number of microbes (Figure 6) and the consumption of nutrients (Figure 7 and Figure 9), thereby attenuating the weight loss of fruit, to a certain extent.

#### 3.2.6. Effects of the SAEW Treatment on the pH Value of Fresh-Cut Apple

The pH of a fruit (i.e., the effective acidity) describes the concentration of hydrogen cations in the fruit and is a common measure of food acidity. The acidity of the fruit not only affects its taste, but also is an indicator of fruit maturity.

**Figure 11:**
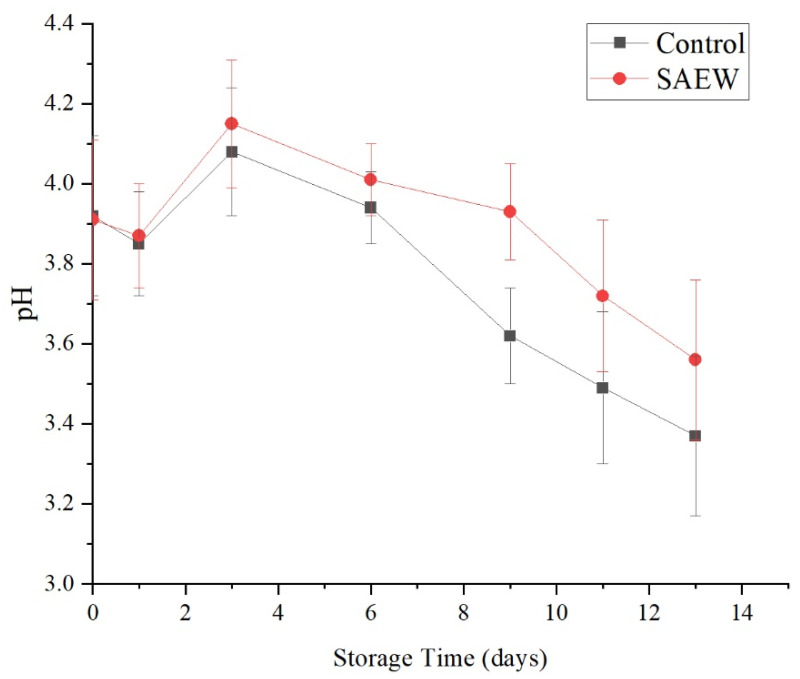
Effect of changes of pH on sample of apples treated with SAEW (4 °C).

As shown in Figure 11, during storage, the pH values of the SAEW-treated and the sterile water-treated groups showed little change, decreasing from 3.91 and 3.92 on d 0 to 3.56 and 3.37 on d 13 of storage. Apart from d 9, there were no significant differences in pH values between the SAEW-treated and control groups on the same days during storage (*p* > 0.05).

Thus, the SAEW treatment had no significant effect on the change in the pH values of fresh-cut apples during storage. This may be related to the organic acid content in fruit. Organic acids are the most readily utilized respiration substrates of fruits. When the amount of microbes is under control, fruit respiration is inhibited, to a certain extent, which reduces the consumption of organic acids [31]. In addition, electrolyzed water influences biofilms and key enzymes in the organism, but has relatively little effect on small-molecule organic acids, resulting in little change in the pH value.

## 4. Conclusions

The SAEW treatment conditions were optimized using a Box–Behnken response surface method, and the resulting optimal conditions are as follows: ACC, 21 mg/L; treatment time, 5min; liquid-to-material, 9:1 mL/g. Under these conditions, the SAEW treatment not only exhibited a satisfactory bactericidal effect on the surface microbes of fresh-cut apples, but also did not adversely affect the apples’ sensory qualities. Moreover, during storage, the SAEW treatment inhibited microbial growth, reduced the declining rates of vitamin C and total sugar contents, slowed the weight loss and browning processes, and maintained the tissues’ pH values, and thus slowed the deterioration of important quality parameters of fresh-cut apples in storage, which extends their shelf life. Thus, the results are valuable for guiding the application of a SAEW during the processing of fresh-cut fruit.

## Figures and Tables

**Table 1:** Coded variables and their coded levels in response surface analysis.

The Variables	Coded	Coded Levels	Levels of Independent		
			−1	0	+1
ACC (mg/L)	A	X_1_	10	20	30
Treatment Time (min)	B	X_2_	3	5	7
Liquid-to-material Ratio (mL/g)	C	X_3_	4:1	8:1	12:1

**Table 2:** The experimental design and results.

No.	ACC A/(mg/L)	Treatment Time B/min	Liquid-to-Material Ratio C/(mL/g)	Microbial Lethality Rate *Y*_1_/lg (CFU/g)	Sensory Score *Y*_2_
1	30	7	8	3.81	7.46
2	20	7	12	3.07	8.44
3	20	5	8	2.93	8.99
4	20	5	8	3.82	8.98
5	20	5	8	3.12	8.99
6	10	7	8	1.61	8.14
7	30	5	4	3.36	7.57
8	30	5	12	3.64	7.67
9	20	5	8	3.08	8.95
10	20	3	4	1.98	8.55
11	10	3	8	1.01	8.42
12	20	5	8	3.06	8.99
13	10	5	12	1.38	8.50
14	30	3	8	3.04	7.59
15	10	5	4	1.47	8.24
16	20	7	4	2.87	8.31
17	20	3	12	2.51	8.70

**Table 3:** Analysis of variance (ANOVA) for regression equation.

Response Value	Source	Sum of Squares	Degree of Freedom	Means Square	*F*-Value	*p*-Value	Significant
Microbial Lethality Rate (*Y*_1_)	Model	11.91	9	1.32	17.29	0.0005	Highly significant
	Residual	0.54	7	0.077			
	Lack of fit	0.038	3	0.013	0.10	0.9547	Not significant
	Pure error	0.50	4	0.12			
	Cor. Total	12.44	16				
R^2^ = 0.9569, R_adj_^2^ = 0.9016							
Sensory Score (*Y*_2_)	Model	4.66	9	0.52	1198.63	<0.0001	Highly significant
	Residual	0.0030	7	0.0004			
	Lack of fit	0.00018	3	0.0006	2.03	0.2526	Not significant
	Pure error	0.0012	4	0.0003			
	Cor. Total	4.66	16				
R^2^ = 0.9994, R_adj_^2^ = 0.9985							

**Table 4:** Significance test for regression coefficients.

Response Value	Coefficients	Regression Coefficient	Degree of Freedom	Standard Error	*F*-Value	*p*-Value
Microbial Lethality Rate (*Y*_1_)	Constant Term	3.20	1	0.12	--	--
	A	8.78	1	8.78	114.70	<0.0001
	B	0.99	1	0.99	12.99	0.0087
	C	0.11	1	0.11	1.38	0.2781
	AB	0.0072	1	0.0073	0.094	0.7676
	AC	0.034	1	0.034	0.45	0.5251
	BC	0.027	1	0.027	0.36	0.5697
	A^2^	1.01	1	1.01	13.20	0.0084
	B^2^	0.50	1	0.50	6.54	0.0377
	C^2^	0.26	1	0.26	3.43	0.1064
Sensory Score (*Y*_2_)	Constant Term	8.98	1	0.0093	--	--
	A	−0.38	1	0.0074	2620.69	<0.0001
	B	−0.11	1	0.0074	239.53	<0.0001
	C	0.080	1	0.0074	118.48	<0.0001
	AB	0.037	1	0.010	13.02	0.0086
	AC	−0.040	1	0.010	14.81	0.0063
	BC	−0.0050	1	0.010	0.23	0.6452
	A^2^	−0.79	1	0.010	6100.09	<0.0001
	B^2^	−0.29	1	0.010	798.36	<0.0001
	C^2^	−0.19	1	0.010	365.76	<0.0001

**Table 5:** Validity verification of the developed regression model.

ACC (mg/L)	Treatment Time (min)	Liquid-to-Material Ratio (mL/g)	Microbial Lethality Rate *Y*_1_		Sensory Evaluation *Y*_2_	
			Predictive Value	Measured Value	Predictive Value	Measured Value
				2.99		8.75
21	5	9:1	3.37	3.14	8.77	8.80
				3.61		8.64

## Data Availability

The data used to support the findings of this study can be made available by the corresponding author upon request.
